# Users’ Intention to Continue Using Online Mental Health Communities: Empowerment Theory Perspective

**DOI:** 10.3390/ijerph18189427

**Published:** 2021-09-07

**Authors:** Jingfang Liu, Jiayu Wang

**Affiliations:** School of Management, Shanghai University, Shanghai 200444, China; jingfangliu@shu.edu.cn

**Keywords:** online mental health communities, empowerment theory, expectation confirmation theory, health self-efficacy theory, continuous intention

## Abstract

Introduction: Online mental health communities may provide new opportunities for rehabilitation for people with mental illness, so it is important to understand the factors that influence the continued use of online mental health communities by people with mental illness. Methods: From the perspective of empowerment, based on the theory of health self-efficacy and expectation confirmation, this study explored the mediating role of health self-efficacy and expectation confirmation in the empowerment process of patients in online mental health communities and users’ intention to continue using online mental health communities. To verify this model, we obtained 272 valid questionnaires. The SmartPLS 3.0 software was selected for model construction and empirical analysis. Results: Health self-efficacy completely mediates the relationship between the empowerment process (i.e., emotional support, information support, helping others and sharing experiences) and users’ intention to continue using an online mental health community. Expectation confirmation partially mediates the relationship between the empowerment process (i.e., information support and finding recognition) and users’ intention to continue using an online mental health community. Conclusion: The empowerment process is the main predictor of user health self-efficacy and expectation confirmation. This study has certain theoretical and practical significance for online mental health community research.

## 1. Introduction

### 1.1. Background

Mental illness is a serious disease that can cause substantial harm to the human body. Depression is a mental illness; extremely depressed people may engage in behaviors that cause physical harm to their bodies. The WHO reported that approximately two-thirds of patients diagnosed with depression do not seek help; that is, the outpatient rate is very low. Therefore, taking no action causes many consequences, including disability, homelessness, unemployment, suicide, inappropriate incarceration, and denial of a full life [1]. Due to the public perception and prejudice against depression, patients with depression are affected by much stigma, which poses a great threat to the physical and mental health of patients with depression. Online mental health communities provide a platform for patients who suffer from depression to share their experiences. These communities empower users to vent their emotions, share their experiences, obtain recognition, and help others [2,3,4]. Online mental health communities are formed spontaneously by patients with mental illness, and uninvited communication among users naturally occurs. This emerging form of social interaction may provide new opportunities for the rehabilitation of patients with mental illness [5] and promote healing of self-esteem and the mental and physical health of people with severe mental illness [6,7,8]. Online mental health communities have been proven to provide the same support as offline support groups, including emotional support, information support, network support, respect support, and to a lesser extent, tangible support [9,10]. These forms of support are common in online mental health communities, especially emotional and information support, and include sharing experiences about treatment and daily life [11,12,13]. Online mental health communities have repeatedly been proven to be effective in eliminating loneliness, breaking down stigma barriers, promoting self-empowerment and encouraging individuals to become more active and informed patients [14]. Challenging stigma, seeking health information and interventions aimed at providing support and promoting physical and mental health have been linked to reducing loneliness and gaining confidence through online interaction with peers [15]. The online mental health community has a large number of users of untreated and undiagnosed patients, which are the target of community interventions (to increase users’ confidence in health awareness and disease management abilities). Communities can provide information and support to curb the stigmatization of traditional information seeking models.

### 1.2. Empowerment Theory

Empowerment theory focuses on the inability of people to meet their needs because of social injustice and discrimination [16]. Therefore, the goal of empowerment is to help people acquire the right to decide and act in their own lives by weakening social or individual obstacles that affect their right to decide and act, enhancing their ability and confidence to use power, deriving certain power from the environment [16]. Empowerment can enable people with mental illness to realize their ideals to obtain happiness [17]. The core concepts of empowerment theory are empowerment, power, powerlessness and social stratification [18]. Empowerment refers to the process by which individuals or groups acquire power resources and control their own lives. Empowerment theory holds that some people in society have no opportunity to acquire power resources and control their own destiny because of the existence of social stratification. Social stratification is attributed to differences in gender, age, race, sexual orientation, health, and other aspects of life. Groups such as women, homosexual people, and people with mental illness face discrimination and unfair treatment [19]. Empowerment has an intrinsic motivational structure that can influence and control the intrinsic impulses of others. People’s needs are met when they realize they have the power or confidence to respond to events and situations. This motivational power manifests itself as self-determined intrinsic needs or self-health efficacy. Therefore, this paper, addresses the importance of the empowerment theory for helping patients with mental illness, a vulnerable group, reduce oppression and improve self-efficacy.

In the context of online health communities, few studies have clearly defined the concept of empowerment. Some studies have focused on the process of patient empowerment in online health communities [20,21,22,23], while others have focused on patient empowerment outcomes [24,25,26,27]. Therefore, based on previous studies on the definition of the empowerment process, this paper clarified the content of the empowerment process of patients in online mental health communities, and explored the impact of the empowerment process on the patients’ beliefs about expectation confirmation and disease management ability. The patient empowerment process in the online mental health community reflects a belief in patient autonomy and the rights and responsibilities of patients to access health information and make health-related decisions [2]. A qualitative study of user-generated content in online patient support groups reveals that the empowerment process in online patient support groups mainly involves an information exchange among patients, individuals obtaining emotional support from other patients, people helping others gain recognition, and individuals sharing experiences and connecting with others [14,23,28]. Through reviewing quantitative and qualitative research on online support for community user behavior, scholars found that the patient users’ self-disclosure, emotional support and information collection in the empowerment process can improve users’ understanding of their own situation, accelerate the development of social relations and improve decision-making skills [14,29]. Scholars have found that the empowerment process of online support groups produces different empowerment results. Respondents cited the following empowering outcomes: access to better information, confidence in their doctors and treatment, improved acceptance of illness, development of optimism, increased control over their emotional and physical conditions, and increased self-esteem and social well-being [4]. Rogers et al. found that empowerment was associated with self-esteem, improved quality of life, social support and satisfaction with mutual assistance programs [19]. Some scholars have found that patient empowerment plays a positive role in improving individual health, combating self-stigma, and promoting the realization of individual goals [30]. Empowerment is strongly associated with most aspects of recovery for people with severe mental illness [17,31]. Research has shown that empowered patients are effective in managing their attitudes toward treatment for their disease and in maintaining their health and access to quality care [32].

Few studies have applied the empowerment process to empirical research, especially to research on users’ intention to use online communities. In previous studies, the author mainly investigated the intention of the continuous use of an online health community from the perspectives of an information system environment and service, personal social cognition, and social influence. For example, Hossain confirmed that the service quality, information quality and interaction quality of online medical websites have significant influences on users’ value perception and satisfaction, thus affecting users’ intention to continue using these websites [33]. Based on the theory of organizational support, Liu et al. explored the influence of online health community support on user interaction and value co-creation and found that both of them had a certain degree of influence on users’ intention to continue using an online health community [34]. However, the process of empowering patients in online health communities had a significant positive effect on patients. Patients have many expectations about the potential empowerment outcomes of participating in online health communities. However, there is no direct evidence that the empowerment process in online mental health communities affects patients, and the empowerment theory has rarely been employed in empirical studies to explore users’ intention to continue using online mental health communities.

### 1.3. Research Questions

Health self-efficacy refers to a person’s belief in their own health management ability [35]. Health self-efficacy is based on the social cognition theory developed by Albert Bandura and is adapted from the original definition that self-efficacy refers to a person’s belief in his or her ability to control and influence his or her life circumstances [36]. Behind the definition of self-efficacy is the principle that a person’s beliefs about their abilities affect how they respond to a given situation. Research has shown that in the field of health, self-efficacy has an important role in the desired outcome of disease. In a study of women with heart disease, self-efficacy was found to be a strong predictor of several disease-management behaviors, including drug use, exercise, eating habits, emotional management, and stress management [37]. Studies have shown that web-based health interventions can significantly improve people’s self-efficacy in certain behaviors, such as physical exercise and a healthy diet, and that physiological and social benefits can positively increase people’s expectations of behavioral change [38]. According to existing studies, self-efficacy is closely related to health-promoting or health-damaging behaviors [39]. In the past, the influence of self-efficacy on the behavior of users in information systems was mainly based on the self-efficacy of users using information systems. Some scholars found that the degree of users’ adaptation to information systems positively affects their attitude towards using these systems [40]. We hypothesized that users’ positive attitudes and beliefs about their own health management had a positive effect on their attitudes toward using an online mental health community.

In research on information systems in the field of health, self-efficacy is mainly utilized as an independent variable [41,42,43,44] or a dependent variable [45]. As previously mentioned, health self-efficacy is not only relatively stable but may also be affected by a large number of other factors in addition to health concerns and social support, which means that health self-efficacy may also be a mediating variable. Few relevant studies have been carried out, so based on previous studies on health information systems and empowerment theory, this study proposes the first research question:

How do the processes involved in patient empowerment in online mental health communities affect patient health self-efficacy? How does the user’s health self-efficacy affect the user’s intention to continue using online mental health communities?

Expectancy confirmation theory is a cognitive theory that attempts to explain satisfaction after purchase or adoption as uncertain factors, such as expectation, perception, and belief. The theory points out that satisfaction is determined by the interaction between expectation before a purchase and evaluation after a purchase [46]. Although expectation confirmation theory was originally applied for the satisfaction of consumers after a purchase, it is now widely employed to investigate the willingness of users to continue using information systems. Online mental health communities empower users. Users engage in community interactions with expectations, such as answers and reassurance. If the user experience is better than expected, then the result is satisfaction. On the other hand, if the user experience meets user expectations, there is a good chance that users will be dissatisfied as a result. This paper interprets the expected confirmation as a positive perception of users’ participation in the online mental health community and is another predictor of their continued use of it. Therefore, the second research question of this paper is explained as follows:

Do the processes involved in patient empowerment in an online mental health community meet the expectations of patient users? Expectation confirmation can affect the user’s intention to continue using online mental health communities.

To answer these research questions, from the perspective of empowerment, this paper constructs a model of a user’s intention to continue using online mental health communities based on health self-efficacy theory and expectation confirmation theory. We assume that each process of empowerment is a predictor of a user’s health self-efficacy and a user’s expected confirmation, and that both health self-efficacy and expectation confirmation have a positive impact on a user’s intention to continue using. To test this model, we conducted a survey.

## 2. Research Hypothesis and Research Model

**Figure 1:**
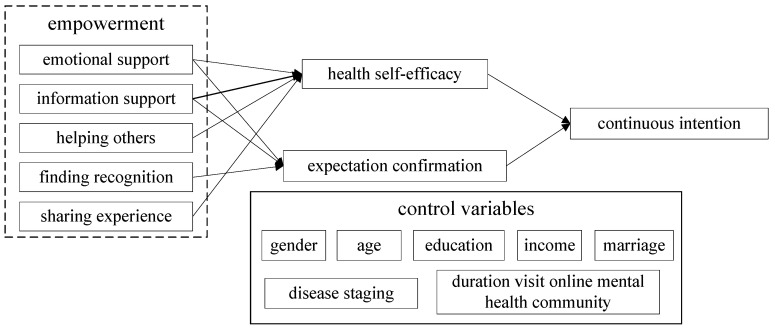
Research model.

To bridge the gap in existing research, to address the limitations of previous studies and to answer the proposed research questions, we developed the model shown in Figure 1. With the health self-efficacy theory and the expectation confirmation theory as the theoretical basis of the model, the various processes of empowerment have a major role. Control variables included gender, age, education, income, marriage, disease staging, and duration of visit to online mental health communities.

The empowerment process of an online health community mainly includes an information exchange, emotional support, helping others, obtaining recognition, sharing experiences, and connecting with others. We can summarize the process of empowerment into five processes: information support, emotional support, helping others, obtaining recognition, and sharing experiences.

Both information exchange and emotional support belong to the category of social support. Social support is a common form of interaction among members in online health communities. Social support among community members contributes to patients’ well-being [34]. Information support and emotional support are very common in the online health community interaction process. The expression form of emotional support can be understood as the expression of sympathy and care for individuals. The primary effect is a psychological sense of comfort, dependence, and belonging for the supported individual. The main form of information support is the feedback of raising questions and opinions for individuals. The main function is to make supported individuals believe that they are cared for, to obtain valuable information from society and to improve the emotional experience and satisfaction of being supported and understood. Through the acquisition of valuable information, patients can enhance their own faith in health management and disease control [47]. Sebastian found that social support and community drivers are important factors for users to actively engage with online commerce sites [48]. There is evidence that people with severe mental illness participate in online support groups and that having peer support can help patients actively participate in treatment and improve disease management and control [49]. Hyun et al. found that health information seeking and emotional support on Facebook had a significant positive impact on users’ health self-efficacy [45]. Therefore, the following hypothesis is proposed:

**Hypothesis** **1.** *Emotional support in online mental health communities positively affects users’ health self-efficacy*.

**Hypothesis** **2.** *Information support in online mental health communities positively affects users’ health self-efficacy*.

**Hypothesis** **3.** *Information support in online mental health communities positively affects users’ expectation confirmation.*

**Hypothesis** **4.** *Emotional support in online mental health communities positively affects users’ expectation confirmation.*

The premise of helping others is to actively provide advice and emotional guidance to other users through the form of language. Riessiman proposed the helper principle in 1965, which states that the helper gains more in the process of helping others [50]. In a study that explores the long-term psychosocial and behavioral effects of helping others control their weight on overweight and previously overweight people, Wallston discovered that people who help others lose weight are significantly more likely to lose weight, are more likely to maintain good eating habits and activity levels, and have better feelings of self and self-control [51]. Individuals, as helpers, share with their peers strategies and resources they discovered to help address life goals that have been hindered by mental illness; these experiences enhance these individuals’ sense of self-efficacy [30]. In this study, users contributed to their belief in their ability to control and manage disease by helping other users in the community, i.e., answering their questions and giving them emotional support. Therefore, the following hypothesis was proposed:

**Hypothesis** **5.** *Helping others positively affects users’ health self-efficacy.*

Obtaining recognition in online mental health communities refers to the notion that other users in the community have similar illnesses and may have similar life experiences, and this similarity enables individuals to feel less lonely, especially for people with mental health problems such as depression. It can be comforting to find others in the community who share the same values and experiences due to social stigma and low self-efficacy [52]. Young people with mental illness report that one of the main reasons for connecting with others online is to feel less lonely [53]. Popular social media allows people with severe mental illness to feel connected to others and to gain a sense of relief from knowing that others are going through similar experiences and challenges [49]. Research has shown that people who develop a positive self-perception through interaction with members of their own group can develop a more positive self-perception. People may be more receptive to information provided by their peers and information that they identify with than other sources of information. In addition, peers can act as positive role models [3,54]. By learning about the illness stories of other participants who acted as positive role models, they became more optimistic about their future. Interviews revealed that participation in online mental health communities allows the participants to temporarily break away from the oppression and tension of the real world and gain tolerance and understanding. The results of participation are satisfactory to them and meet their expectations. Therefore, the following hypotheses are proposed:

**Hypothesis** **6.** *Finding recognition positively affects the user’s expectation confirmation.*

Sharing experiences in the process of empowerment mainly refers to recording a person’s own experience of disease treatment in the community, including diagnosis and medication. In addition to health-related experiences, sharing some daily life experiences, including daily trifles and emotional changes, are also part of the process [5]. Some scholars have found that sharing emotions online is significantly related to the relief of patients’ negative emotions [55]. Sharing personal experiences online is a form of self-disclosure. In real life, due to social stigma, people with mental illness seldom talk about their illness with others. However, online mental health communities increase opportunities for self-disclosure [56,57]. The increased confidence and belonging gained by selectively revealing their illness to others online may even make it easier for people to disclose their condition in face-to-face encounters [58]; thus, they have more confidence in their own disease management and control. Sharing strategies and resources with peers whom with they have connected to help address life goals that have been hindered by mental illness is a surrogate experience for other members that enhances an individual’s health self-efficacy [30]. Sharing experience means that destinations strategically educate people about mental illness. In special groups with similar conditions for sharing mental illness in the past and present experience, sharing experiences has an added benefit over indiscriminate disclosure, that is, it has trained a strength against mental illness and stigma [59,60]. Therefore, the following hypothesis is proposed:

**Hypothesis** **7.** *Sharing experiences positively affects users’ health self-efficacy.*

Health self-efficacy refers to the degree to which users feel that an online health community helps them build confidence in overcoming difficulties in the diagnosis and treatment process. Self-efficacy is a key factor in recovering from mental health disorders [61]. Users’ perceived usefulness to an online health community has a significant positive impact on their willingness to continue using the community [62]. By participating in an online mental health community, users perceive their ability to improve health management and disease control and will naturally have a positive attitude towards the continued use of the online mental health community. Based on the health self-efficacy established in this paper, it is believed that users’ beliefs about their ability to manage their health are important factors influencing users’ continued use of an online mental health community, and the following hypotheses are proposed:

**Hypothesis** **8.** *Health self-efficacy positively affects the intention to continue using online mental health communities.*

Expectancy confirmation is when a person’s perception of an outcome conforms to a set of expectations. Expectancy confirmation theory states that satisfaction is directly influenced by expectation confirmation [63]. Users are satisfied when the post-use experience meets their expectations. If a user is satisfied, he or she will have a stronger intention to continue using an online mental health community. The positive correlation between community satisfaction and continued use has been confirmed by previous research. For example, the user’s intention to continue using an information system is determined by the individual’s satisfaction and perceived usefulness [64]. Therefore, expectation confirmation has a positive impact on intention to continue use. So, we propose the following hypothesis:

**Hypothesis** **9.** *Expectations confirmation positively affects users’ intention to continue using online mental health communities.*

All constructs used in the model are shown in Table 1.

## 3. Research Methodology

### 3.1. Research Context

Online mental health communities are spontaneously formed by patients with mental diseases; consider depression online communities as an example, whose main users are patients with depression. Users can participate in an online mental health community by viewing posts in various sections of the community, commenting on posts, and liking or forwarding posts. Users can also post messages seeking help from other users in the community, such as information, social and emotional support. The online mental health community allows users to vent in the form of a post and encourages patients to share their treatment experiences and record their lives. Most of the users in the community are patients suffering from depression. Although the causes of their illness may differ, they suffer from the same mental illness and have similar symptoms and needs; this similarity makes them a special social group. By using the online mental health community as a medium, they communicate with each other and support each other, which has a positive effect on their own disease management, cognitive emotion and coping with depression and promotes the recovery of the mental and physical health of patients with severe mental illness. The research object of this paper is an online depression community, which has 283,000 users and ranks first on the medical topic list. Therefore, examining the continuous use intention has great value.

### 3.2. Measurement

The construct items were based on previous studies, with adjustments to fit the specific research context. All items were measured on a 5-point Likert-type scale, ranging from strongly disagree (one) to strongly agree (five). The construction items are shown in Table 2. The reliability and validity of the measurement model are analyzed, and then the hypothesis of the structural model ability is verified.

### 3.3. Data Collection

The questionnaire was targeted at users of an online mental health community. A total of 293 questionnaires were collected from June to July 2021 through a combination of online questionnaires (Questionnaire Star) and online interviews. According to the trap questions and abnormal answer times, invalid questionnaires were excluded, and all questionnaires with the same answers were deleted. A total of 272 valid questionnaires were obtained. The effective recovery rate was 92.8%. Demographic data were also collected for each respondent in the survey. Demographic data for the respondent are shown in Table 3. Institutional review board approval was obtained for this study.

## 4. Data Analysis and Results

As shown in Table 3, the respondents were mainly female, and their age was concentrated in the teenage stage.

In terms of the data analysis, first we analyzed the reliability and two kinds of validity (i.e., convergent validity and discriminant validity). Second, the structural model was evaluated, and the assumptions were tested. As some of the structures and relationships investigated in the research model are new, the research is exploratory. In our study, SmartPLS was used for measurement and structural model analysis. when the purpose of the study is exploratory, this method is feasible [65].

### 4.1. Measurement Model

In the model test, a confirmatory factor analysis was performed to test the measurement model, including the internal consistency reliability test and convergence validity test. Reliability was measured by composite reliability (CR) and Cronbach’s alpha (Cronbach’s α) values, and the validity was extracted by the average variance extracted (AVE) by the square of each potential variable and corresponding measurable variable load. The results are shown in Table 4. The CR values of all potential variables are greater than 0.7, and Cronbach’s α values are greater than 0.7, indicating a good reliability of the sample data. The AVE values of all variables are greater than 0.5, indicating good convergence validity of the sample data [66,67,68].

Discriminant validity is a measure of the degree of difference between two different constructs [66,69]. Based on previous studies, discriminative validity was tested by comparing the square root of the AVEs and the correlation between this variable and other variables. As shown in Table 5, we determined that all square roots of AVE are higher than the correlations among the variables. Therefore, the discriminant validity is acceptable, and our measurement model is reliable.

### 4.2. Structural Model

To test the impact of health self-efficacy and expectation confirmation, we compared the least-squares regression models of the three models; the results are shown in Table 6. In Model 1, only control variables were added. The results showed that control variables explained 8.4% of the variance of dependent variables. Only age (*β* = −0.161, *p* < 0.05) had a significant negative effect on users’ continued use of online health communities, while the other control variables had no significant effect on users’ continued use intention.

In Model 2, information support, emotional support, obtaining recognition and expectation confirmation were also included. The results showed that information support (*β* = 0.27, *p* < 0.001) and finding recognition (*β* = 0.243, *p* < 0.001) had a positive effect on expectation confirmation. H3 and H6 were supported. However, emotional support (*β* = 0.074, *p* > 0.05) had no significant effect on users’ expectation confirmation. The inclusion of information support, emotional support, and obtaining recognition and expectation confirmation increased the R-square value from 0.084 to 0.265.

In Model 3, five variables, including emotional support, information support, helping others, sharing experience and health self-efficacy, were added based on Model 1. The results showed that emotional support (*β* = 0.323, *p* < 0.001), information support (*β* = 0.184, *p* < 0.01), helping others (*β* = 0.16, *p* < 0.05), and sharing experience (*β* = 0.191, *p* < 0.01) had significant positive effects on health self-efficacy. H1, H2, H5, and H7 were supported. Healthy self-efficacy (*β* = 0.796, *p* < 0.001) had a significant positive impact on continued use intentions in online mental health communities. H8 is supported. Compared with Model 1, *R*^2^ changed from 0.084 to 0.668.

In order to understand the essential relationship among various processes of patient empowerment, health self-efficacy and intention to continue using, this paper added a mediating effect test. This test is common in behavioral sciences to design models for mediating variables [70]. In the mediation model, there is not a direct causal relationship between the independent variable and the dependent variable but rather that the independent variable affects the intermediate variable, which affects the dependent variable. Therefore, the role of the intermediate variable is to clarify the nature of the relationship between the independent variable and the dependent variable [70,71].

**Figure 2:**
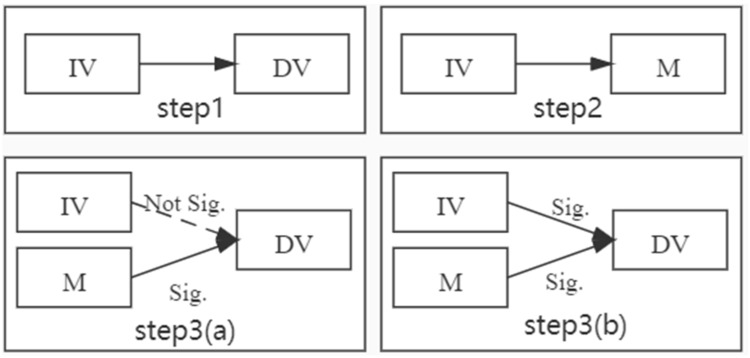
Mediation test steps. Note 1: IV: Independent variable; M: mediator; DV: dependent variable. A → B means regress A on B (i.e., A has influence to B). Note 2: Step 1, IV → DV is significant. Step 2: IV → M is significant. Step 3: IV + M → DV. (**a**) If M is significant and IV is not significant, then M fully mediates the effect of IV on DV. (**b**) If both M and IV are significant, then M partially mediates the effect of IV on DV [71].

This paper used the method proposed by Baron and Kenny [72] to examine mediating effects. This method has three steps. The first step is the regression of the dependent variable to the independent variable (IV→DV). The second step is the regression of the independent variable (IV→M) with the intermediate variable. In the third step, the dependent variable regresses the intermediate variable and the independent variable (IV + M→DV). In step 3, if M is significant but IV is not, then M completely mediates the effect of IV on DV. If both M and IV are significant, M partially mediates the effect of IV on DV. The mediation test method is shown in Figure 2.

As shown in Table 7, health self-efficacy completely mediates the relationship between the empowerment process (i.e., emotional support, information support, helping others and sharing experiences) and users’ intention to continue using the online mental health community. Each process of user empowerment has no significant influence on users’ intention to continue using.

## 5. Research Analysis and Discussion

### 5.1. Key Findings

The study had several main findings. First, emotional support, information support, helping others and sharing experiences in the empowerment process have significant positive effects on users’ health self-efficacy, which indicates that the abovementioned four empowerment processes can help users improve their ability to control and manage their own diseases in the process of participating in the online mental health community.

Second, the improvement of health self-efficacy can promote users’ intention to continue using online mental health communities. If users’ self-efficacy is improved, their enthusiasm for disease treatment will be enhanced. Users perceive that participating in an online mental health community is beneficial to their disease management ability, which promotes their continuous use of the online mental health community.

Third, information support and recognition have a positive impact on users’ expectation confirmation, indicating that the health information provided by online mental health communities and the information exchanged among users meets users’ needs. An environment where members of an online mental health community connect and understand each other is a good for people with mental health problems. Emotional support had no significant effect on expectation confirmation, which may be attributed to the notion that depressed people experience mood swings. Therefore, emotional support among the members of an online community does not truly relieve the user’s depression but only strengthens the user’s confidence to fight the disease, which cannot degenerate.

Fourth, expectation confirmation has a positive effect on the willingness to continue using an online mental health community. As online mental health communities discourage discrimination and exclusion, users can be authentic and openly express themselves. Therefore, meeting the user’s expectations coupled with learning much about the disease can motivate the user to continue to use online mental health communities.

### 5.2. Theoretical Contribution

This study provides several theoretical contributions.

First, this paper studies the intention to continue using online mental health communities from the perspective of empowerment. The findings indicate the empowerment process (i.e., emotional support, information support, helping others and sharing experiences) has a significant positive effect on the user’s health self-efficacy. This outcome establishes a new perspective for future research.

Second, health self-efficacy acted as a mediating variable in this paper, reflecting the essential relationship between the empowerment process of online mental health communities and users’ intention to continue using; health self-efficacy had a complete mediating role in this. This finding establishes a new way of thinking and research direction for the study of user cognition in an online health community.

### 5.3. Implications

The results have several practical implications.

First, research shows that emotional supports, information support, helping others and sharing experiences in the empowerment process have a significant positive impact on users’ health self-efficacy. The improvement of health self-efficacy means the improvement of patients’ beliefs in their own health management and disease control ability, which is an omen of the improvement in patients’ condition. This finding has implications for the online mental health community, which can conduct interventions. For example, the community can often disseminate knowledge about the treatment of diseases and principles of diseases and can add a life record feature to encourage users to share and record. Positive articles, heartfelt songs, interesting stories, etc., can be shared regularly to make users feel connected, thus enhancing user engagement.

Second, for professional mental health care personnel, online mental health communities can improve people’s health self-efficacy to a certain extent, so professionals can appropriately encourage patients to actively participate in online mental health communities.

In addition, the most important aspect is geared toward the patients. By participating in online mental health communities, patients can encourage others, record moments in life, and express themselves openly in the community. Someone comforts them when they feel sad, shares in their happiness, and provides support when they need it, thus enhancing their happiness and worth. Through sharing the experiences of others, participants can enhance their confidence in disease treatment and develop a positive outlook on life and their disease.

### 5.4. Limitations and Future Research

The sample employed in this study is limited. The sample objects obtained in this study focus on women in the teenage stage, but depression spans a large age group. Therefore, in the future, subjects of all ages can be selected from multiple online mental health communities. Such research results are more convincing, and differentiation analysis among different samples can be completed.

## 6. Conclusions

In this paper, we examine a meaningful research question about the intention to continue using online mental health communities. This research comprises the study of the user’s intention to continue using from the perspective of empowerment. Based on health the self-efficacy theory and the expectation confirmation theory, a model of online mental health users’ intention to continue using was established. It was found that emotional support, information support, helping others and sharing experiences in the empowerment process significantly affected users’ health self-efficacy and that health self-efficacy had a significant positive impact on users’ intention to continue using online mental health communities. Health self-efficacy completely mediates the relationship between the empowerment process (i.e., emotional support, information support, helping others and sharing experiences) and users’ intention to continue using an online mental health community. Expectation confirmation partially mediates the relationship between the empowerment process (i.e., information support and obtaining recognition) and users’ intention to continue using an online mental health community.

The study found that helping others and sharing experiences in online mental health communities had a positive effect on the improvement in users’ health self-efficacy, indicating that users can benefit from their own health management and disease control by helping others and actively expressing and sharing, which provided some inspiration for online mental health communities. Community managers can design more boards for users to express their ideas according to different themes to encourage users to share, record, and promote more positive experiences in disease treatment and popularize knowledge of related diseases. Positive articles, heartfelt songs, interesting stories, etc., can be shared regularly to make users feel connected, thus enhancing user engagement. Health care professionals can appropriately encourage people with mental illness to actively participate in online mental health community discussions.

## Figures and Tables

**Table 1:** Summarization of constructs used in the research model.

Constructs	Definition	Source
Emotional support	Receiving encouragement, comfort, and understanding from others	[45]
Information support	Receiving information about health	[45]
Helping others	Providing information and emotional support to others	[2]
Finding recognition	Feeling accepted and connected	[2]
Sharing experiences	Sharing experiences of treatment and daily life	[2]
Health self-efficacy	Belief in their ability to manage health and control disease	[35]
Expectation confirmation	Perception of the outcome is consistent with established expectations	[64]
Continuous intention	Intention to continue using online mental health communities	[64]

**Table 2:** Constructs and items used in the questionnaire.

Constructs		Items	Literature
Emotional support	ES1	Provide encouragement to me	[45]
	ES2	Show me empathy	
	ES3	Make me feel relieved	
Information support	IS1	Ask for health information or treatment advice	[45]
	IS2	Search and find a lot of health information	
	IS3	The exchange of health information and advice meets the need	
	IS4	Add extra value to the information I obtain from my doctor	
Helping others	HO1	I can offer advice and support to others	[2]
	HO2	I can be an example to other participants	
Finding recognition	FR1	I recognize myself in the stories of others	[2]
	FR2	I experience the sense of ‘not being the only one’	
	FR3	Others are an example to me	
	FR4	I realize that I am not so bad off after all	
Sharing experiences	SE1	I can share my experiences with my illness with others	[2]
	SE2	I can share my everyday experiences with others	
Expectation confirmation	EC1	My experience with the community has been better than I expected	[64]
	EC2	The service provided by the community was better than I had expected	
	EC3	The community can meet demand beyond my expectations	
Health self-efficacy	SEF1	I am confident that I can make a positive impact on my health	[45]
	SEF2	I have set some definite goals to improve my health	
	SEF3	I am taking active treatment to improve my health	
Continuous intention	CI1	I will continue to be involved in this community in the future	[35]
	CI2	I will be coming back to this community often	
	CI3	I will recommend others with depression be involved in this community	

**Table 3:** Demographic data of respondents.

Variables	Value	Frequency	Percentage
Gender	male	37	13.6
	female	235	86.4
Age	<18	87	32
	18–29	176	64.7
	30–40	8	2.9
	>40	1	0.4
Marriage	unmarried	263	96.7
	married	9	3.3
Education	Junior or less	48	17.6
	Senior high school	80	29.4
	Junior college	45	16.5
	Undergraduate	92	33.8
	Postgraduate	7	2.6
Income	<1000	198	72.8
	1000–3000	27	9.9
	3001–5000	23	8.5
	5001–8000	16	5.9
	8001–15,000	6	2.2
	>15,000	2	0.7
Disease staging	Mild depressed	63	23.2
	Moderate depressed	94	34.6
	Severe depressed	107	39.3
	Cured	8	2.9
Duration visit online mental health community	<10 min	73	26.8
	10 min–30 min	140	51.5
	30 min–1 h	40	14.7
	>1 h	19	7

**Table 4:** Confirmatory factor analysis.

Variables	Items	Standard Loads	Cronbach’s α	CR	AVE
Emotional support	ES1	0.887	0.833	0.9	0.75
	ES2	0.847			
	ES3	0.863			
Information support	IS1	0.806	0.879	0.917	0.734
	IS2	0.879			
	IS3	0.859			
	IS4	0.881			
Helping others	HO1	0.961	0.905	0.954	0.913
	HO2	0.95			
Finding recognition	FR1	0.754	0.793	0.862	0.61
	FR2	0.762			
	FR3	0.808			
	FR4	0.799			
Sharing experiences	SE1	0.937	0.85	0.93	0.869
	SE2	0.927			
Health self-efficacy	SEF1	0.872	0.772	0.868	0.688
	SEF2	0.829			
	SEF3	0.785			
Expectation confirmation	EC1	0.9	0.903	0.939	0.837
	EC2	0.929			
	EC3	0.916			
Continuous intention	CI1	0.933	0.873	0.923	0.799
	CI2	0.905			
	CI3	0.842			

CR: Composite reliability; AVE: average variance extracted.

**Table 5:** Discriminant validity results of the measurement model.

	CI	EC	ES	FR	HO	IS	SE	SEF
CI	**0.894**							
EC	0.447	**0.915**						
ES	0.472	0.371	**0.866**					
FR	0.463	0.411	0.61	**0.786**				
HO	0.374	0.303	0.421	0.559	**0.955**			
IS	0.485	0.428	0.608	0.58	0.473	**0.862**		
SE	0.377	0.31	0.434	0.584	0.569	0.502	**0.932**	
SEF	0.815	0.394	0.586	0.545	0.492	0.566	0.512	**0.829**

Note: The diagonal numbers in bold are the square roots of the AVEs.

**Table 6:** PLS results.

Variables	Model 1	Model 2	Model 3
Gender	–0.077 (0.059)	−0.085 (0.049)	−0.063 (0.042)
Age	−0.161 * (0.077)	−0.124 (0.065)	−0.086 (0.044)
Education	−0.046 (0.072)	−0.077 (0.068)	−0.005 (0.043)
Income	−0.055 (0.072)	−0.035 (0.059)	0.011 (0.034)
Marriage	0.018 (0.07)	0.046 (0.05)	0.007 (0.026)
Depression degree	−0.017 (0.063)	−0.008 (0.057)	−0.02 (0.045)
Duration visit online support group	−0.026 (0.072)	0.043 (0.065)	−0.053 (0.046)
Emotional support		0.074 (0.062)	0.323 *** (0.057)
Information support		0.27 *** (0.064)	0.184 ** (0.067)
Helping others			0.16 * (0.07)
Finding recognition		0.243 *** (0.058)	
Sharing experience			0.191 ** (0.068)
Health self-efficacy			0.796 *** (0.049)
Confirmation		0.433 *** (0.056)	
Observations	272	272	272
*R* ^2^	0.084	0.265	0.668

*Note*: The numbers are the beta values. The numbers in parentheses are standard errors. *** *p* < 0.001; ** *p* < 0.01; * *p* < 0.05.

**Table 7:** Results of the mediation effects test.

IV	M	DV	IV—>DV	IV—>M	IV + M—>DV		Mediating
					IV—>DV	M—>DV	
ES	SEF	CI	0.480 ***	0.594 ***	−0.006	0.822 ***	completely
IS	SEF	CI	0.480 **	0.558 *	0.037	0.798 ***	completely
HO	SEF	CI	0.387 ***	0.497 ***	−0.032	0.834 ***	completely
SE	SEF	CI	0.388 ***	0.518 **	−0.054	0.846 ***	completely

*Note*: *** *p* < 0.001; ** *p* < 0.01; * *p* < 0.05.

## References

[1] Lawlor A., Kirakowski J. (2014). Online support groups for mental health: A space for challenging self-stigma or a means of social avoidance?. Comput. Hum. Behav..

[2] Uden-Kraan C., Drossaert C., Taal E., Seydel E.R., van de Laar M.A.F.J. (2009). Participation in online patient support groups endorses patients’ empowerment. Patient Educ. Couns..

[3] Uden-Kraan C.V., Drossaert C., Taal E., Shaw B.R., Seydel E.R., van de Laar M.A.F.J. (2008). Empowering processes and outcomes of participation in online support groups for patients with breast cancer, arthritis, or fibromyalgia. Qual. Health Res..

[4] Mo P.K., Coulson N.S. (2014). Are online support groups always beneficial? A qualitative exploration of the empowering and disempowering processes of participation within HIV/AIDS-related online support groups. Int. J. Nurs. Stud..

[5] Ziebland S., Wyke S. (2012). Health and Illness in a Connected World: How Might Sharing Experiences on the Internet Affect People’s Health?. Milbank Q..

[6] Lehman A.F., Kernan E., Deforge B.R., Dixon L. (1995). Effects of homelessness on the quality of life of persons with severe mental illness. Psychiatr. Serv..

[7] Folsom D.P., Hawthorne W., Lindamer L., Gilmer T., Jeste D.V. (2005). Prevalence and risk factors for homelessness and utilization of mental health services among 10,340 patients with serious mental illness in a large public mental health system. Am. J. Psychiatry.

[8] Pompili M., Lester D., Innamorati M., Tatarelli R., Girardi P. (2008). Assessment and treatment of suicide risk in schizophrenia. Expert Rev. Neurother..

[9] Coursaris C.K., Ming L. (2009). An analysis of social support exchanges in online HIV/AIDS self-help groups. Comput. Hum. Behav..

[10] McCormack A. (2010). Individuals With Eating Disorders and the Use of Online Support Groups as a Form of Social Support. CIN Comput. Inform. Nurs..

[11] Uden-Kraan C., Drossaert C., Taal E., Seydel E.R., van de Laar M.A.F.J. (2008). Self-Reported Differences in Empowerment Between Lurkers and Posters in Online Patient Support Groups. J. Med. Internet Res..

[12] Malik S.H., Coulson N.S. (2011). A Comparison of Lurkers and Posters Within Infertility Online Support Groups. CIN Comput. Inform. Nurs..

[13] Winzelberg A. (1997). The analysis of an electronic support group for individuals with eating disorders. Comput. Hum. Behav..

[14] Prescott J., Rathbone A.L., Brown G. (2020). Online peer to peer support: Qualitative analysis of UK and US open mental health Facebook groups. Digit. Health.

[15] Naslund J.A., Aschbrenner K.A., Marsch L.A., Bartels S.J. (2016). The future of mental health care: Peer-to-peer support and social media. Epidemiol. Psychiatr. Sci..

[16] Conger J.A., Kanungo R.N. (1998). The empowerment process. Integration theory and practice. Acad. Manag. J..

[17] Corrigan P.W., Giffort D., Rashid F., Leary M., Okeke I. (1999). Recovery as a Psychological Construct. Community Ment. Health J..

[18] Rogers E.S., Chamberlin J., Ellison M.L., Crean T. (1997). A consumer-constructed scale to measure empowerment among users of mental health services. Psychiatr. Serv..

[19] Rogers E.S., Ralph R.O., Salzer M.S. (2010). Validating the empowerment scale with a multisite sample of consumers of mental health services. Psychiatr. Serv..

[20] Jerry F. (1999). An Exploration of Helping Processes in an Online Self-Help Group Focusing on Issues of Disability. Health Soc. Work.

[21] Klemm P., Bunnell D., Cullen M., Soneji R., Gibbons P., Holecek A. (2003). Online cancer support groups: A review of the research literature. Comput. Inform. Nurs..

[22] Perron B. (2002). Online support for caregivers of people with a mental illness. Psychiatr. Rehabil. J..

[23] Sharf B.F. (1997). Communicating breast cancer on-line: Support and empowerment on the Internet. Women Health.

[24] Broom A. (2005). Virtually Healthy: The Impact of Internet Use on Disease Experience and the Doctor-Patient Relationship. Qual. Health Res..

[25] Hill W., Weinert C., Cudney S. (2006). Influence of a computer intervention on the psychological status of chronically ill rural women: Preliminary results. Nurs. Res..

[26] Hoybye M.T., Johansen C. (2009). Online interaction. Effects of storytelling in an internet breast cancer support group. Psycho-Oncology.

[27] Powell J., Mccarthy N., Eysenbach G. (2004). Cross-sectional survey of users of Internet depression communities. BMC Psychiatry.

[28] Mo P.K.H., Coulson N.S. (2010). Empowering processes in online support groups among people living with HIV/AIDS: A comparative analysis of ‘lurkers’ and ‘posters’. Comput. Hum. Behav..

[29] Barak A., Boniel-Nissim M., Suler J. (2008). Fostering empowerment in online support groups. Comput. Hum. Behav..

[30] Corrigan P.W., Larson J.E., Ruesch N. (2013). Self-stigma and the “why try” effect: Impact on life goals and evidence-based practices. World Psychiatry.

[31] Corrigan P.W., Mark S., Ralph R.O., Yvette S., Lorraine K. (2004). Examining the Factor Structure of the Recovery Assessment Scale. Schizophr. Bull..

[32] Mo P.K., Coulson N.S. (2012). Developing a model for online support group use, empowering processes and psychosocial outcomes for individuals living with HIV/AIDS. Psychol. Health.

[33] Hossain M.A. (2016). Assessing m-Health success in Bangladesh: An empirical investigation using IS success models. J. Enterp. Inf. Manag..

[34] Liu W., Fan X., Ji R., Jiang Y. (2019). Perceived Community Support, Users’ Interactions, and Value Co-Creation in Online Health Community: The Moderating Effect of Social Exclusion. Int. J. Environ. Res. Public Health.

[35] Sun Y.L., Hwang H., Hawkins R., Pingree S. (2008). Interplay of Negative Emotion and Health Self-Efficacy on the Use of Health Information and Its Outcomes. Commun. Res..

[36] Bandura A. (1977). Self-Efficacy: Toward a Unifying Theory of Behavioral Change. Psychol. Rev..

[37] Clark N.M., Dodge J.A. (1999). Exploring Self-Efficacy as a Predictor of Disease Management. Health Educ. Behav..

[38] Andersonbill E.S. (2011). Social Cognitive Determinants of Nutrition and Physical Activity Among Web-Health Users Enrolling in an Online Intervention: The Influence of Social Support, Self-Efficacy, Outcome Expectations, and Self-Regulation. J. Med. Internet Res..

[39] Pálsdóttir A. (2008). Information behaviour, health self-efficacy beliefs and health behaviour in Icelanders’ everyday life. Inf. Res..

[40] Rana N.P., Dwivedi Y.K. (2015). Citizen’s adoption of an e-government system: Validating extended social cognitive theory (SCT). Gov. Inf. Q..

[41] Mo X., Deng Z. (2015). Analysis of the Influence of Health Self-efficacy on Health Information Adoption via SNS. Chin. J. Health Stat..

[42] Peng Y., Deng Z., Wu J. (2019). Analysis of Knowledge Sharing Behavior of Medical Professional Users in Online Health Communities Based on Social Capital and Motivation Theory. Data Anal. Knowl. Discov..

[43] Wu T.L., Deng Z.H., Feng Z.C., Gaskin D.J., Zhang D.L., Wang R.X. (2018). The Effect of Doctor-Consumer Interaction on Social Media on Consumers’ Health Behaviors: Cross-Sectional Study. J. Med. Internet Res..

[44] Imlawi J., Gregg D. (2020). Understanding the satisfaction and continuance intention of knowledge contribution by health professionals in online health communities. Inform. Health Soc. Care.

[45] Oh H.J., Lauckner C., Boehmer J., Fewins-Bliss R., Li K. (2013). Facebooking for health: An examination into the solicitation and effects of health-related social support on social networking sites. Comput. Hum. Behav..

[46] Oliver R.L. (1980). A Cognitive Model of the Antecedents and Consequences of Satisfaction Decisions. J. Mark. Res..

[47] Mittal V.A., Tessner K.D., Walker E.F. (2007). Elevated social Internet use and schizotypal personality disorder in adolescents. Schizophr. Res..

[48] Molinillo S., Anaya-Sánchez R., Liébana-Cabanillas F. (2020). Analyzing the effect of social support and community factors on customer engagement and its impact on loyalty behaviors toward social commerce websites. Comput. Hum. Behav..

[49] Naslund J.A., Grande S.W., Aschbrenner K.A., Elwyn G. (2014). Naturally occurring peer support through social media: The experiences of individuals with severe mental illness using YouTube. PLoS ONE.

[50] Frank R. (1965). The “Helper” Therapy Principle. Soc. Work.

[51] Wallston K.A., Mcminn M., Katahn M., Pleas J. (1983). The helper-therapy principle applied to weight management specialists. J. Community Psychol..

[52] Harvey K.J., Brown B., Crawford P., Macfarlane A., McPherson A. (2007). ‘Am I normal?’ Teenagers, sexual health and the internet. Soc. Sci. Med..

[53] Burns J.M., Durkin L.A., Nicholas J. (2009). Mental health of young people in the United States: What role can the internet play in reducing stigma and promoting help seeking?. J. Adolesc. Health.

[54] Turner G. (1999). A method in search of a theory: Peer education and health promotion. Health Educ. Res..

[55] Liu X., Pan M., Li J. (2019). Does Sharing Your Emotion Make You Feel Better? An Empirical Investigation on the Association Between Sharing Emotions on a Virtual Mood Wall and the Relief of Patients’ Negative Emotions. Telemed. E-Health.

[56] Kim J., Lee J.E. (2011). The Facebook paths to happiness: Effects of the number of Facebook friends and self-presentation on subjective well-being. Cyberpsychol. Behav. Soc. Netw..

[57] Bazarova N.N., Choi Y.H. (2014). Self-Disclosure in Social Media: Extending the Functional Approach to Disclosure Motivations and Characteristics on Social Network Sites. J. Commun..

[58] Mckenna K., Bargh J.A. (1998). Coming out in the age of the Internet: Identity “demarginalization” through virtual group participation. J. Personal. Soc. Psychol..

[59] Whitley R., Campbell R.D. (2014). Stigma, agency and recovery amongst people with severe mental illness. Soc. Sci. Med..

[60] Bargh J.A., Mckenna K. (2004). The internet and social life. Annu. Rev. Psychol..

[61] Castelein S., van der Gaag M., Bruggeman R., van Busschbach J.T., Wiersma D. (2008). Measuring Empowerment Among People With Psychotic Disorders: A Comparison of Three Instruments. Psychiatr. Serv..

[62] Wu B. (2018). Patient Continued Use of Online Health Care Communities: Web Mining of Patient-Doctor Communication. J. Med. Internet Res..

[63] Oliver R.L. (1977). Effect of expectation and disconfirmation on postexposure product evaluations: An alternative interpretation. Psychol. Rep..

[64] Weber R. (2001). Understanding information systems continuance: An expectation-confirmation model. MIS Q..

[65] Wynne C.W. (1998). Issues and Opinion on Structural Equation Modeling. MIS Q..

[66] Mcknight D.H., Choudhury V., Kacmar C. (2002). Developing and Validating Trust Measures for e-Commerce: An Integrative Typology. Inf. Syst. Res..

[67] Anderson J., Gerbing C., David W. (1988). Structural equation modeling in practice: A review and recommended two-step approach. Psychol. Bull..

[68] Komiak S.Y.X., Benbasat I. (2006). The Effects of Personalization and Familiarity on Trust and Adoption of Recommendation Agents. MIS Q..

[69] O’Leary-Kelly S.W., Vokurka R.J. (1998). The empirical assessment of construct validity. J. Oper. Manag..

[70] Preacher K.J., Hayes A.F. (2004). SPSS and SAS procedures for estimating indirect effects in simple mediation models. Behav. Res. Methods Instrum. Comput..

[71] Bhattacherjee A., Hikmet N. (2007). Physicians’ resistance toward healthcare information technology: A theoretical model and empirical test. Eur. J. Inf. Syst..

[72] Baron R.M., Kenny D.A. (1986). The moderator-mediator variable distinction in social psychological research: Conceptual, strategic, and statistical considerations. Chapman Hall.

